# Brain Damage in Commercial Breath-Hold Divers

**DOI:** 10.1371/journal.pone.0105006

**Published:** 2014-08-12

**Authors:** Kiyotaka Kohshi, Hideki Tamaki, Frédéric Lemaître, Toshio Okudera, Tatsuya Ishitake, Petar J. Denoble

**Affiliations:** 1 Center for Hyperbaric Medicine and Environmental Health, University Hospital of the Ryukyus, Nishihara, Okinawa, Japan; 2 Tamaki Hospital, Hagi, Yamaguchi, Japan; 3 Faculty of Sport Sciences, University of Rouen, Mont-Saint-Aignan, France; 4 Department of Neuro-radiology and Psychiatry, Shin-funagoya Hospital, Miyama, Fukuoka, Japan; 5 Department of Environmental Medicine, Kurume University School of Medicine, Kurume, Fukuoka, Japan; 6 Divers Alert Network, Durham, North Carolina, United States of America; National Research Council of Italy, Italy

## Abstract

**Background:**

Acute decompression illness (DCI) involving the brain (Cerebral DCI) is one of the most serious forms of diving-related injuries which may leave residual brain damage. Cerebral DCI occurs in compressed air and in breath-hold divers, likewise. We conducted this study to investigate whether long-term breath-hold divers who may be exposed to repeated symptomatic and asymptomatic brain injuries, show brain damage on magnetic resonance imaging (MRI).

**Subjects and Methods:**

Our study subjects were 12 commercial breath-hold divers (Ama) with long histories of diving work in a district of Japan. We obtained information on their diving practices and the presence or absence of medical problems, especially DCI events. All participants were examined with MRI to determine the prevalence of brain lesions.

**Results:**

Out of 12 Ama divers (mean age: 54.9±5.1 years), four had histories of cerebral DCI events, and 11 divers demonstrated ischemic lesions of the brain on MRI studies. The lesions were situated in the cortical and/or subcortical area (9 cases), white matters (4 cases), the basal ganglia (4 cases), and the thalamus (1 case). Subdural fluid collections were seen in 2 cases.

**Conclusion:**

These results suggest that commercial breath-hold divers are at a risk of clinical or subclinical brain injury which may affect the long-term neuropsychological health of divers.

## Background

Decompression illness (DCI), which includes bubble disease from decompression sickness and arterial gas embolism is well known in compressed-air divers. The common symptoms are join and/or muscle pain, numbness, or paresthesia, whereas stroke-like syndromes are uncommon [1]. In contrast, the occurrence of DCI in breath-hold diving is considered extremely rare [2]. However, several cases of DCI have been reported in professional and sport breath-hold divers [3], [4], [5], [6]. Our survey of commercial breath-hold divers (Ama) in Japan have shown that repetitive, working breath-hold dives can induce neurological DCI. Based on the reported symptoms and neuro-radiological findings, brain lesions were particularly prominent in this group [7], [8].

Numerous studies have been conducted in an attempt to evaluate the long-term neuropsychological consequences of professional and recreational compressed air diving. It was shown that incidence of brain lesions increases with diving experience, suggesting that diving could have a cumulative effect on the brain even without a history of symptomatic injuries [9], [10]. Moreover, similar concerns regarding subclinical cerebral lesions without a history of symptomatic DCI have emerged recently in compressed air divers [11]. On the other hand, breath-hold divers with a known history of stroke-like post-dive symptoms have not been studied using neuro-radiological methods [7], [8], so far. The purpose of the present study was, therefore, to assess the prevalence of brain injury in Japanese Ama divers, using by brain magnetic resonance imaging (MRI).

## Subjects and Methods

Twelve male Ama divers from two villages on the archipelagos Aishima and Ohshima, members of the diving union of Abu-Hagi district of Yamaguchi Prefecture, Japan, participated in the study. In one village there were 13 partially assisted Ama divers and six volunteered for the study. In the other village there were six partially assisted Ama divers and all six participated in the study. No specific medical selection criterion was applied. The Ama diving union includes 14 villages of divers on archipelagos and on the coast of the main island. In April 2009, there were 346 male and 35 female divers; 39 of them were assisted and were all male [8]. Their age ranges from their teens to 80 s. Unassisted Ama divers generally dive to depths of 3–6 meters without any aids. Assisted divers use weights for descents and are either pulled up by assistants (completely assisted) or swim up without assistance (partially assisted). The assisted divers work deep, occasionally over 20 meters [11]. In the present study all participants were partially assisted Ama divers.

All assisted and unassisted Ama divers engage in daily diving work during harvest season (from December 21 to October 20); Monday through Thursday. In accordance with their regulations, diving starts at 09∶00 and stops at 15∶00. They wear wetsuits and fins and carry a weight belt to achieve neutral buoyancy. They work two shifts a day with a lunch break of about 30 minutes in between. The duration of the morning shift was three to four hours and that of the afternoon shift was one to three hours. They descend about 30 times per hour to depths of 10–20 meters [12]. Their working dive pattern has not been changed for a few decades.

The study protocol was approved by the human research ethics committee of Kurume University School of Medicine, Japan. The subjects participated voluntarily and gave their written informed consent. In 2011 a standard interview was carried out by a physician (H.T.), focusing on a diving history including possible diving-related symptoms and neurological findings such as joint pain, skin rash, nausea, dizziness/vertigo, motor and/or sensory involvement, visual deficit, and unconsciousness. Diving accidents were defined as event with symptom onset within 24 hours after dive [1]. A separate questionnaire for the divers included questions on their smoking habit, alcohol consumption, and the presence of hypertension, cardiac disease, diabetes mellitus and cerebrovascular diseases.

The participants were enrolled into the brain MRI study beginning in 2011 using the following criteria: 1) partially assisted Ama divers, 2) no contraindications to MRI, and 3) signed informed consent. We made axial brain images (T1: TR/SE 220/400 msec, T2: TR/SE 220/4000 msec, slice thickness 9 mm) on a 0.2 Tesla MRI scanner (AIRIS mate, Hitachi, Japan) at Tamaki hospital, Hagi city, Yamaguchi Prefecture in Japan. We defined chronic cerebral infarcts as areas of focal hyperintensity on T2-weighted images that were at least 3 mm in diameter. Areas of hyperintensity also had to have corresponding prominent hypointensity on T1-weighted images. In addition, white-matter lesions were considered present if they were shown as hyperintense on T2-weighted images, without prominent hypointensity on T1-weighted scans. However, lesions in the supraventricular and paraventricular white matter were distinguished. MR films were reviewed jointly and without blinding, in conference by a neuro-radiologist (T.O.) and a neurosurgeon (K.K.).

## Results

Significant findings obtained from the participants are shown in **Table 1**. The details of diving exposure were as follows; total Ama diving experience of 29.8±7.6 years (range: 17–40 years) and partially assisted Ama experience of 26.3±10.1 years (range: 9–40 years). Seven of 12 Ama divers began to work as unassisted divers in shallow water, and then graduated to become partially assisted divers. Other divers started as assisted Ama divers from their begging. The divers carried a weight belt of 5±1.5 kg (range: 1–7 kg) to maintain neutral buoyancy, and assisted Ama divers descended passively using a weight of 20.4±1.5 kg (range: 18–24 kg) and swam to the surface without assistance.

**Table 1:** Physical and medical characteristics and brain MRI lesions in 12 Ama divers.

case/age	tabacco, pack-year	alcohol, g/day	career of Ama, years	medical history	history of diving accidents	Significant MRI findings
1/58	–	90	24	No	No	cortex-subcortex (1), white matter (1)
2/49	30	45	17	No	No	cortex (3)
3/44	22	50	21	No	No	negative
4/48	–	0	30	diabetes	hemiparesis, speech disturbance	subcortex (1)
5/59	20	0	27	No	No	cortex (1), subcortex (1)
6/56	–	0	21	angina	No	putamen (1)
7/61	–	90	38	No	sensory numbness	cortex (1), putamen (2), putaminal hemorrhage (1)
8/61	–	90	36	diabetes	No	subcortex (1), white matter (2)
9/54	–	90	30	No	No	cortex (1), white matter (2), thalamus (2)
10/56	–	90	40	No	No	cortex (3), putamen (1)
11/58	80	25	40	No	hemiparesis	white matter (1), caudate nucleus (2), subdural effusion, enlargement of ventricle
12/55	36	0	34	No	sensory numbness	cortex (1), subdural effusion

*: parenthesis: numbers of lesion.

Four of 12 Ama divers had experienced stroke-like neurological events during or immediately after multiple breath-hold dives; their symptoms were attributable to brain insults and all of them were transient and resolved completely within a few hours (No. 4, 7, 11, 12) **(Table-1)**. The time intervals between the last neurological events and the MRI study were more than several years in these divers. Two divers had history of two decompression neurological accidents. One of them (No. 4) had experienced speech disturbance and motor weakness on right side after 3–4 hours repetitive dives, and the symptoms disappeared spontaneously within 1 hour after the accident. Moreover, his former event included transient hemiparesis involving facial paresis around 10 years before. The medical histories included diabetes mellitus in two, and angina in one diver.

Eleven of 12 Ama divers displayed MRI abnormalities of the brain **(Table-1)**. All ischemic lesions were supratentorial, predominantly in the cortex and/or subcortex (9 cases), central white matter (4 cases), basal ganglia (4 cases), and thalamus (1 case). There were two lesions close to the lateral ventricles in one diver, which would show infarcts in the caudate nucleus, not primary demyelinating disorders. In addition, questionable areas on MRI suggesting cortical infarcts or subacute stage of infarction were seen in five cases (No. 1, 2, 4, 5, 6). Other brain lesions were subdural effusion in two (No. 11, 12), and lateral ventricle enlargement caused by white matter infarction in one diver (No. 11).

**Figure 1:**
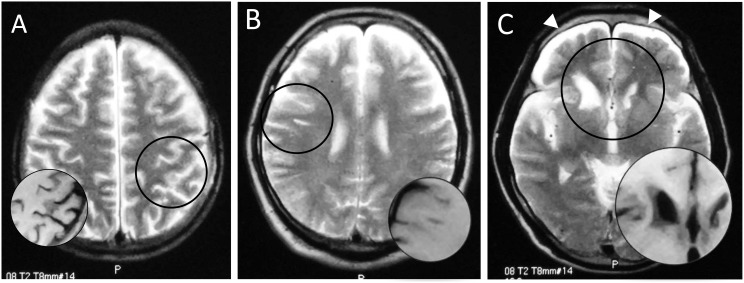
Magnetic resonance images of brains of three Ama divers: hyperintense area on T2-weighted image (circle), corresponding to hypointensity on T1-weighted image (inset). A patchy shadow in the left parietal cortex (A, No. 2), a linear subcortical lesion in the right frontal lobe (B, No. 5), and deformity of bilateral caudate heads and subdural fluid collection (allow heads) (C, No. 11).

**Figure 1** shows examples of MRI from three divers who were found to have brain lesions. Two divers had no history of neurological DCI events (Fig. 1A & B, No. 2 & 5) and the other one had transient left-sided hemiparesis after multiple dives for several hours (Fig. 1C, No. 11).

## Discussion

To our knowledge, the present study is the only series in the literature to report the results of brain MRI in commercial breath-hold divers. In our small series of 12 Ama divers, all the participants except for one diver had multiple cerebral infarcts, while they were neurologically normal and healthy divers though four of them had a history of transient stroke-like attacks. Although the used type of low-field MR imager, a popular system at local hospitals in Japan, might have some limitations, its diagnostic quality in detecting cerebral lesions is not different than that of high-field MRI [13]. These results suggest that long-term breath-hold diving increases a risk for brain damage even in the absence of neurological DCI events.

A variety of neuro-radiological studies performed with brain MRI comparing populations of compressed-air divers with non-diving control groups has given conflicting results [9], [10], [14], [15]. Gas bubbles formed in the venous blood after diving, can cross from venous side to the arterial side of the circulation (arterialization) in the presence of intracardiac right-to-left shunts (RLSs). Arterialized gas emboli have been considered one of the main causes of decompression-related pathology. Recently, Gempp et al. demonstrated that the proportion of hyperintense spots in compressed-air divers was closely related to the presence of a large RLS [16], as suggested by Knauth et al [17]. The presence of RLSs in divers is an important risk factor in cerebral DCI events. However, a few reported Ama cases showed no RLSs even though they had large cerebral infarcts [5], [18]. A prevalence rate of RLSs is 10–30% in healthy adults [19], [20]. Local hyperintense areas of the brain observed with MRI are common in healthy subjects, and the prevalence increases with older age and occurs in 10–20% of people aged around 60 years [21], [22]. The high prevalence of cerebral ischemic lesions that we found in Ama divers cannot be explained by RLSs and aging togather.

Based on the reported cases and series, neurological DCI in breath-hold diving appears to exist as a clinical entity [3], [4], [5], [6], [7], [8]. A survey for 173 Ama divers in a district of Japan showed that 12 divers had histories of acute neurological events. Eleven of 29 assisted and only one of 144 unassisted Ama divers had an event. The events were significantly correlated with a severity of dive exposure, i.e. dive depth, dive time, and surface interval [8]. Unlike in compressed air diving, in multiple deep breath-hold diving the cerebral insults appear to be common. The neurological DCI in breath-hold divers is limited to the brain [4], [6], [7], [8], while sparing the spinal cord, which, in contrast, occur more frequently in compressed-air divers. Typically, the cerebral DCI manifests with sensory numbness or motor weakness on one side. Another characteristic of DCI in breath-hold divers is that many divers exhibited transient neurological deficits that resolve within several hours, even without treatments [7], [8].

The cerebral ischemic lesions in Ama divers were predominantly located in the cortex and subcortical white matter; suggested features of circulatory disturbance at the corticomedullary junctional area of cerebral arteries. Other lesions in the basal ganglia were situated in the terminal zone, and the lesions involving central white matter corresponded to border zone or watershed areas. These MRI findings in Ama divers are so-called low-flow cerebral infarction as a result of low perfusion pressure in terminal supply areas and in agreement with those in compressed-air divers [23], [24], [25]. In general, the main clinical features of infarction in these areas of the brain are transient ischemic attacks or mild, even though the underlaying lesions appear considerably large on CT or MRI [26]. Cerebral arterial gas embolization typically involves the migration of gas to small arteries (average diameter, 30 to 60 micron) in terminal supply areas [27], and the size of lesions is mainly influenced by numbers of gas emboli and the clinical course would be dependent on the locations of cerebral lesions.

The potential mechanisms of brain involvement following multiple breath-hold dives are still unclear. In compressed-air diving, nitrogen (N2) dissolves and accumulates in tissues, particularly in fat tissues. Due to possible N2 accumulation after multiple dives with short surface intervals, N2 bubbles are formed in the venous side of tissues rather than the brain. Although the bubble formation has been controversial in breath-hold diving, our recent study on the same 12 participants showed signs of intravascular bubbles using Doppler ultrasound after continuous diving for 3 hours in one diver [12]. Moreover, Matsuo et al. reported a case of an Ama diver with an early stage of acute neurological DCI whose brain CT scan demonstrated an air density in the cortical area, explanatory for his symptoms [18]. These results suggest that multiple breath-hold dives are sufficient to cause N2 retention in divers and produce intravasucular bubbles.

Although a strong correlation between high levels of intravascular bubbles and the risk of DCI is well known in compressed air diving [28], cerebral insults are rare compared to spinal disorders. In contrast, the neurological DCI events in breath-hold divers seem to be due to brain involvement alone despite the difficulty of detecting venous bubbles. The discrepancy between presence of symptoms of cerebral DCI in absence of strong evidence of intravascular bubbles in breath-hold diving has not been resolved, and reviews of the published images suggest that several mechanisms may be at play. All venous bubbles except for micro-bubbles after multiple breath-hold dives are retained or trapped in the small pulmonary arteries. When breath-hold divers descend to the bottom, the trapped bubbles are compressed and decreased enough to pass through the pulmonary capillaries into arterial circulation. The arterialized bubbles expand during ascent, and then cause a small-vessel disease of the brain. While some other mechanisms have been also discussed, in our opinion, cerebral DCI cases in breath-hold divers may be explained by arterial gas embolism as the main mechanism.

While the exact clinical significance of hyperintense lesions is uncertain at this time, their presence is generally considered an intermediate marker for risks of disturbed neuro-psychological performance. Considerable research has been performed to evaluate the potential long-term effects of diving-related DCS on mental health in breath-hold divers.

In conclusion, our results suggest that long-term commercial breath-hold diving can cause brain damages even if neurological DCI events were transient or have not occurred in divers.
